# Rice-Straw Mulch Reduces the Green Peach Aphid, *Myzus persicae* (Hemiptera: Aphididae) Populations on Kale, *Brassica oleracea* var. *acephala* (Brassicaceae) Plants

**DOI:** 10.1371/journal.pone.0094174

**Published:** 2014-04-08

**Authors:** Reinildes Silva-Filho, Ricardo Henrique Silva Santos, Wagner de Souza Tavares, Germano Leão Demolin Leite, Carlos Frederico Wilcken, José Eduardo Serrão, José Cola Zanuncio

**Affiliations:** 1 Departamento de Biologia Animal, Universidade Federal de Viçosa, Viçosa, Brazil; 2 Departamento de Fitotecnia, Universidade Federal de Viçosa, Viçosa, Brazil; 3 Instituto de Ciências Agrárias, Universidade Federal de Minas Gerais, Montes Claros, Brazil; 4 Departamento de Proteção Vegetal, Universidade Estadual Paulista, UNESP, campus of Botucatu, Botucatu, Brazil; 5 Departamento de Biologia Geral, Universidade Federal de Viçosa, Viçosa, Brazil; Centro de Investigación y de Estudios Avanzados, Mexico

## Abstract

Organic mulches, like peel and rice-straw, besides other materials affect the UV and temperature, which cause a reduction in the aphid arrival. The aim was to evaluate the effect of covering the soil with straw on the populations of the green peach aphid, *Myzus persicae* on the kale, *Brassica oleracea* var. *acephala* plants. The first experiment evaluated the direct effect of the rice-straw mulch and the second its indirect effect on aphid immigration, testing the plant characteristics that could lead to the landing preference of this insect. The third experiment evaluated the direct effect of the mulch on the aphid population. In the second and third experiments, four plants, each in a 14 L polyethylene pot with holes at the bottom, were used in areas with and without soil mulching. These pots were changed between areas, after seven days, to evaluate the effects of this change on the arrival of the winged aphids to the plants. Each plant was covered with anti-aphid gauze and inoculated with one winged *M. persicae*. Winged and apterous adults of this insect were counted per plant after 15 days. The temperature increased in the mulched plots to a maximum of 21–36°C and to 18–32°C in the plots with or without soil covering, respectively. Plant growth reduced the numbers of the winged aphids landing before and after they were moved to the bare soil plots. The nutrient content was similar in plants in both the mulched and no mulched plots. The population growth of *M. persicae* was higher in the control than in the mulched plots. This was partially due to temperatures close to 30°C in these plots and changes in the plant physiology. The soil mulching with rice-straw decreased the *M. persicae* landing, increased the plot temperatures and improved the vegetative growth of the kale plants.

## Introduction

The chemical control of pests on the *Brassica* spp. L. (Brassicaceae) plants increased the production costs and environment contamination [1], [2]. However, reflective mulches, including rice-straw, *Oryza sativa* L. (Poaceae) is a suitable alternative to reduce it [3]–[5]. Mulch with synthetic materials and rice peels or rice-straw can reduce the aphid infestation [3], [6], [7]. The organic and synthetic mulches can repel the winged aphid due to theirs color, heat reflection and increase in the UV and air temperature near to the mulch, although synthetic ones are more reflective [3], [8].

The peel and rice-straw reflect UV, which reduced the virus transmitted by the cabbage aphid, *Brevicoryne brassicae* L., 1758 (Hemiptera: Aphididae) to potato, *Solanum tuberosum* L. (Solanaceae) and kale, *Brassica oleracea* var. *acephala* (Brassicaceae) plants [4], [9]. This covering is more significant than the synthetic ones, as it is more economical and does not require removal [10]–[12], improves the soil by incorporating organic material and does not contaminate the environment [13]–[15]. Mulch can also directly affect the aphid immigration and their populations indirectly by changes in the plant physiology which could lead to the lower landing preference and population growth of these insects [16]–[18].

The green peach aphid, *Myzus persicae* Sulzer, 1776 (Hemiptera: Aphididae) is one of the most important agricultural pests in the world, which reduces the yield production in more than 40 different plant families [19], [20]. This peach aphid is an efficient vector of more than 100 plant viruses [21], [22] and resistant to the major insecticides being used [23], [24].

This work studied the effects of the rice-straw mulch on the *M. persicae* populations on kale plants, to test the following hypotheses: (a) the mulch reduces the aphid landing on the kale plants; (b) this effect is due to the higher air temperature in the plant environment, (c) plant nutrient content changes, and (d) the factors that increase aphid population.

## Materials and Methods

### The Direct Effect of the Mulch on Aphid Immigration

This experiment evaluates the direct effect of the rice-straw mulch on the *M. persicae* immigration to the kale plants in the field at the “Universidade Federal de Viçosa (UFV)” in Viçosa, Brazil. The kale clone cultivar (*B. oleraceae* var. *acephala*) was obtained from the germplasm bank of the UFV and the rice-straw mulch from the agro-ecological sector of the UFV. The experiment was conducted with five randomized blocks and two treatments (with or without the mulch). Each plot had 16 plants spaced at 0.80 m×0.80 m and 1.20 m apart.

The kale seedlings were then transplanted and the plants were thinned on October 16 and 17, 2012 leaving them with five leaves each. A total of 2.5 L of chicken manure and 200 g of 4-14-8 NPK were applied per hole during the transplantation of the kale plants and they were daily sprinkler-watered over the next 15 days.

Winged and apterous aphids were removed from the kale leaves and 1.056 Kg of dry matter.m^–2^ of rice-straw per hectare was placed on the soil. The winged aphids landing on the four central plants per plot were counted and removed daily at 5∶00 P.M. between October 18 and November 1, 2012. The four central plants were chosen to avoid edge effects on the lateral ones. The daily maximum temperature was recorded with a max-min thermometer in the center of the plots at plant canopy height, opposed to direct sunlight. The maximum temperature was recorded because it was positively correlated to the aphid flying period [25], [26].

The data were evaluated by covariance analysis, with Poisson error and χ2 of Pearson to correct over dispersion, following F test at 5% probability. The average winged *M. persicae* which landed on the four kale plants per plot were obtained over 15 days. The maximum average temperature was the first exploratory variable (x_1_), the mulched and no mulched treatments, the second one (x_2_), and the mean number of the winged aphids the response one (y) on plots sampled. “Sistema para Análises Estatísticas e Genéticas (SAEG)” version 9.1 [27] (Supplier: UFV) was the software used for data analysis.

### The Indirect Effect of the Mulch on Aphid Immigration

The second experiment evaluated the indirect effect of the mulch on aphid immigration to test if the effect on the nutrient content of the kale plants was correlated with the aphid landing. These plants were cultivated in pots in bare soil or in mulch plots and their positions were changed after seven days. The change in the positions of the pots was made after the evaluations: those in the plots with soil covering were transferred to plots without covering and vice-versa. Counting and removal of the winged aphids continued for more seven days. The lower degree of immigration of this insect to the bare soil plots after changing the pots positions would imply that the plants carried over some characteristics from the previous environment. The kale seedlings were transplanted on April 30, 2013 to 14 L polyethylene pots with soil fertilized with 50 g of NPK (formulation 4-14-8) and 2.0 L of chicken manure which was chosen due to its richness in nutrients and availability [28]. Poultry manure may have some additional effects on aphid population along with effects of rice-straw mulch such as increase plant vigor and water retention [29]. All the aphids were eliminated and the pots were placed into holes at ground level in the field on May 30, 2013. The plot location and design, leaf thinning, sampling, aphid removal, rice-straw layer and irrigation were conducted similar to the first experiment.

The aphids were counted from May 30 to June 5, 2012 (‘beginning’ period) and from June 6 to June 12, 2012 (latter period) after the pots had their positions changed. Two leaves were taken from a kale plant per plot in June 5, 2012 to determine their macronutrient content. The material sampled was washed with deionized water, dried until constant weight, grounded and the NO_3_^–^, NH_4_^+^, P, K, Ca, Mg and S content were determined based on the dry matter [30], [31].

Data were evaluated by multiple regression analysis, with Poisson error, and χ2 Pearson to correct over dispersion followed by the F test at 5% probability. The average daily number of winged *M. persicae* on the plants per plot was obtained during the seven day period. The exploratory variables were x_1_ = NO_3_^–^, x_2_ = NH_4_^+^, x_3_ = P, x_4_ = K, x_5_ = Ca, x_6_ = Mg, x_7_ = S and x_8_ = mulch treatments and the mean number of winged aphids was the result (y) on the plots sampled. The nutrient content of the kale plants on the mulched and bare soils was compared with the analysis of variance (p≤0.05).

### The Effect of Mulch on the Aphid Population Growth

One winged *M. persicae* was inoculated per kale leaf to evaluate the mulch effect on the growth of the aphid colony. These leaves were covered with anti-aphid gauze to prevent the inoculated aphids from flying out, other aphids from landing and to avoid predation. The aphids were inoculated on June 12, 2012 and all the nymphs and winged adults of *M. persicae* were counted on June 27, 2012. This experiment had five replications, and the plot design and plant manipulation were similar to the second experiment.

The data were evaluated using the variance analysis with Poison error and F test at 5% probability. The treatments (with or without mulch) were considered the exploratory variable (x_1_) and the number of nymphs and adult winged aphids were the response variables (y).

No specific permits are required for the plantation of kale and rearing *M. persicae* in Brazil. The field and laboratory studies did not involve endangered or protected species.

## Results

**Figure 1:**
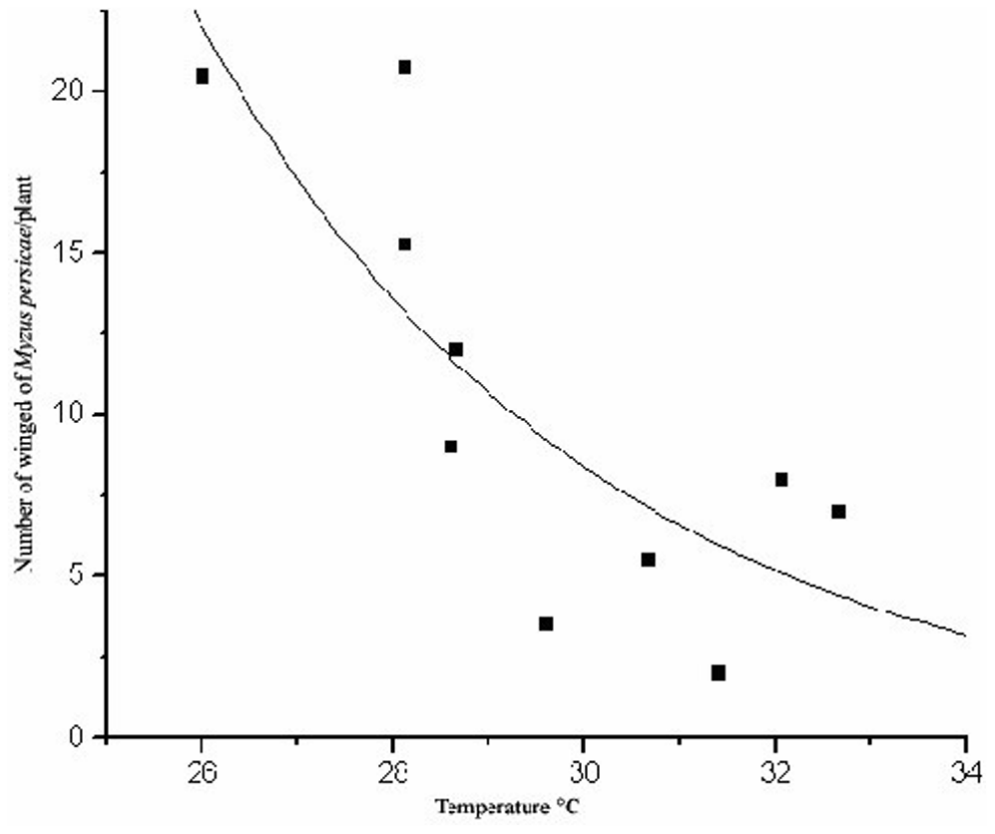
Number of winged green peach aphid, *Myzus persicae* (Hemiptera: Aphididae) landed on kale, *Brassica oleracea* var. *acephala* (Brassicaceae) plants as related to maximum temperature in the plots, with F_1,9_ = 39.03, p≤0.008, r^2^ = 0.60. Each dot corresponds to the mean number of aphids per plant in 10 plots with a Poisson error and χ2 of Pearson to correct over dispersion, followed F test at 5% probability.

**Figure 2:**
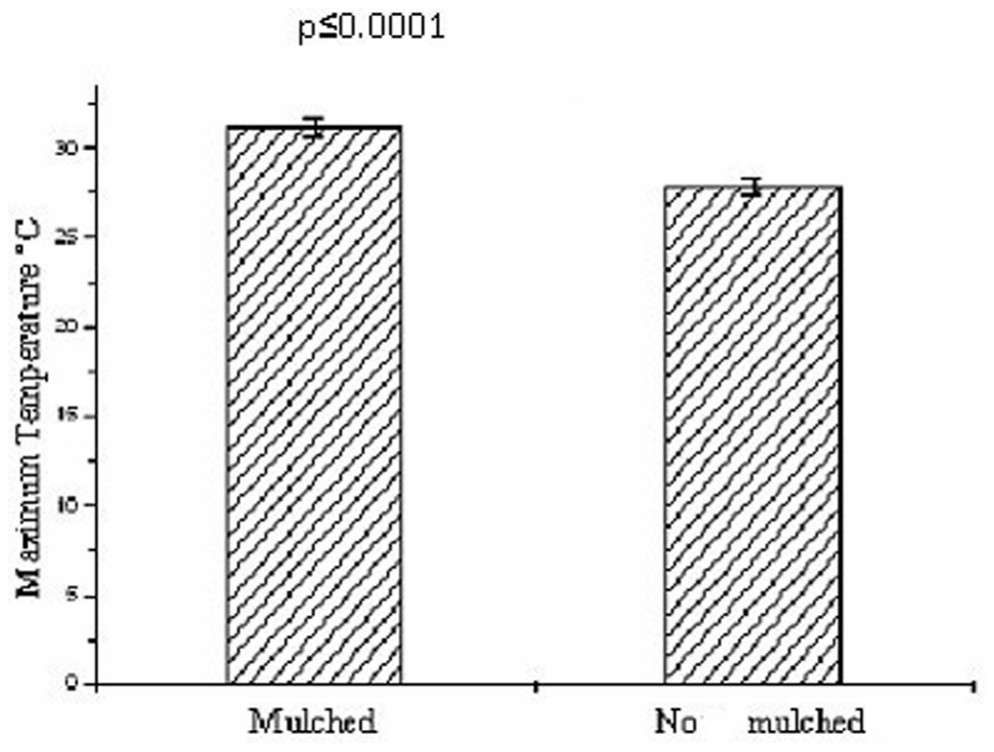
Maximum temperature in the plots with kale, *Brassica oleracea* var. *acephala* (Brassicaceae) plants on mulched and no mulched plots (average of 15 days) with Poisson error and χ2 of Pearson to correct over dispersion followed by an F test at 5% probability.

The number of winged *M. persicae* was lower on the kale plants of the mulched plots than on those in the bare soil (F = 39.03; d.f. = 1.8; p≤0.008), 83 and 327 individuals, respectively, during the monitoring period. The mulch reduced the number of the winged *M. persicae* on the kale plants during the 15-day period (Fig. 1) and increased the maximum temperature at canopy height (Fig. 2). The maximum temperature ranged between 21–36°C and 18–32°C on the mulched and no mulched plots, respectively, which implies that these factors had reduced the arrival of the winged aphids.

**Figure 3:**
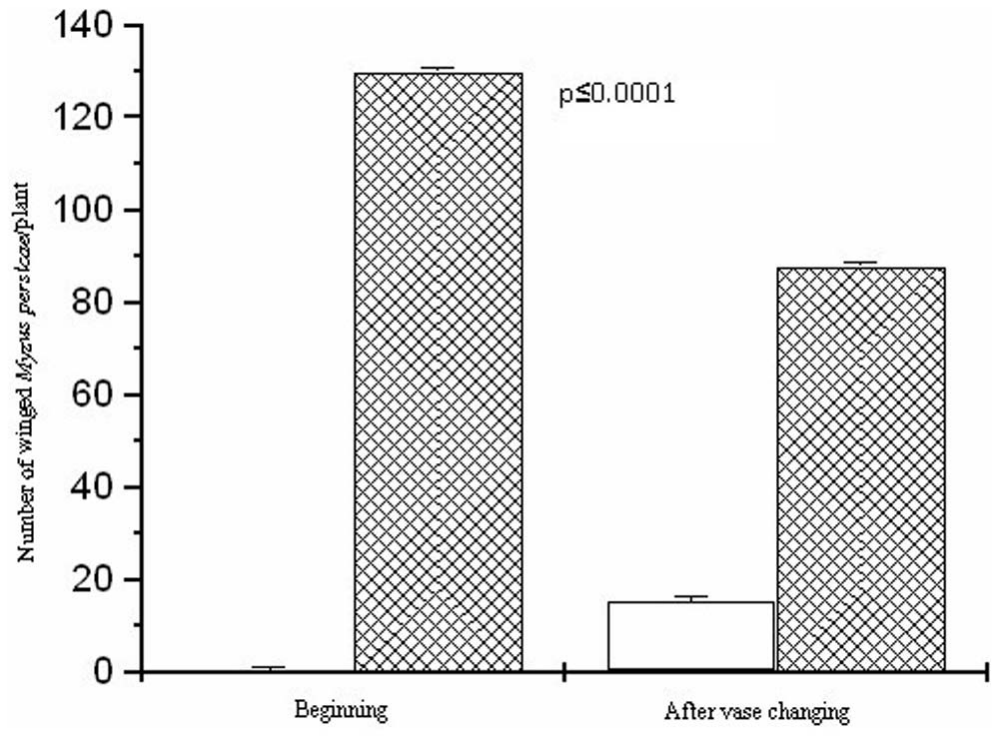
Mean number of winged green peach aphid, *Myzus persicae* (Hemiptera: Aphididae) landed on kale, *Brassica oleracea* var. *acephala* (Brassicaceae) plants on mulched and no mulched plots (hatched bars) at the beginning (days 1–7) and after vase position was changed (days 8–14). Poisson error and χ2 of Pearson to correct over dispersion followed by an F test at 5% probability.

As in the first experiment, the mulch decreased the *M. persicae* immigration (F = 26.52; d.f. = 1.7; p≤0.0001). The number of individuals of this aphid was 1 and 61 and 520 and 351, at the beginning and after the change in the positions of the pots, in the mulched and no mulched plots, respectively. The number of aphids which landed was higher in the no mulched plants moved to the mulched plots (F = 26.52; d.f. = 1.7; p≤0.0001), compared with the ‘beginning’ period. On the other hand, plants from the mulched plots had a lower number of winged aphids when moved to the no mulched plots (Fig. 3). The macronutrient content of the kale leaves was similar in the mulched and no mulched plots (p≥0.05), with means of: NO_3_^–^ = 1.30 dag.Kg^–1^, NH_4_^+^ = 1.36 dag.Kg^–1^, Ca = 1.88 dag.Kg^–1^, S = 0.10 dag.Kg^–1^, K = 2.41 dag.Kg^–1^, Mg = 0.43 dag.Kg^–1^, and P = 0.46 dag.Kg^–1^. The nutrient content boron no relation to the number of aphids on the kale plants (p≥0.05).

**Figure 4:**
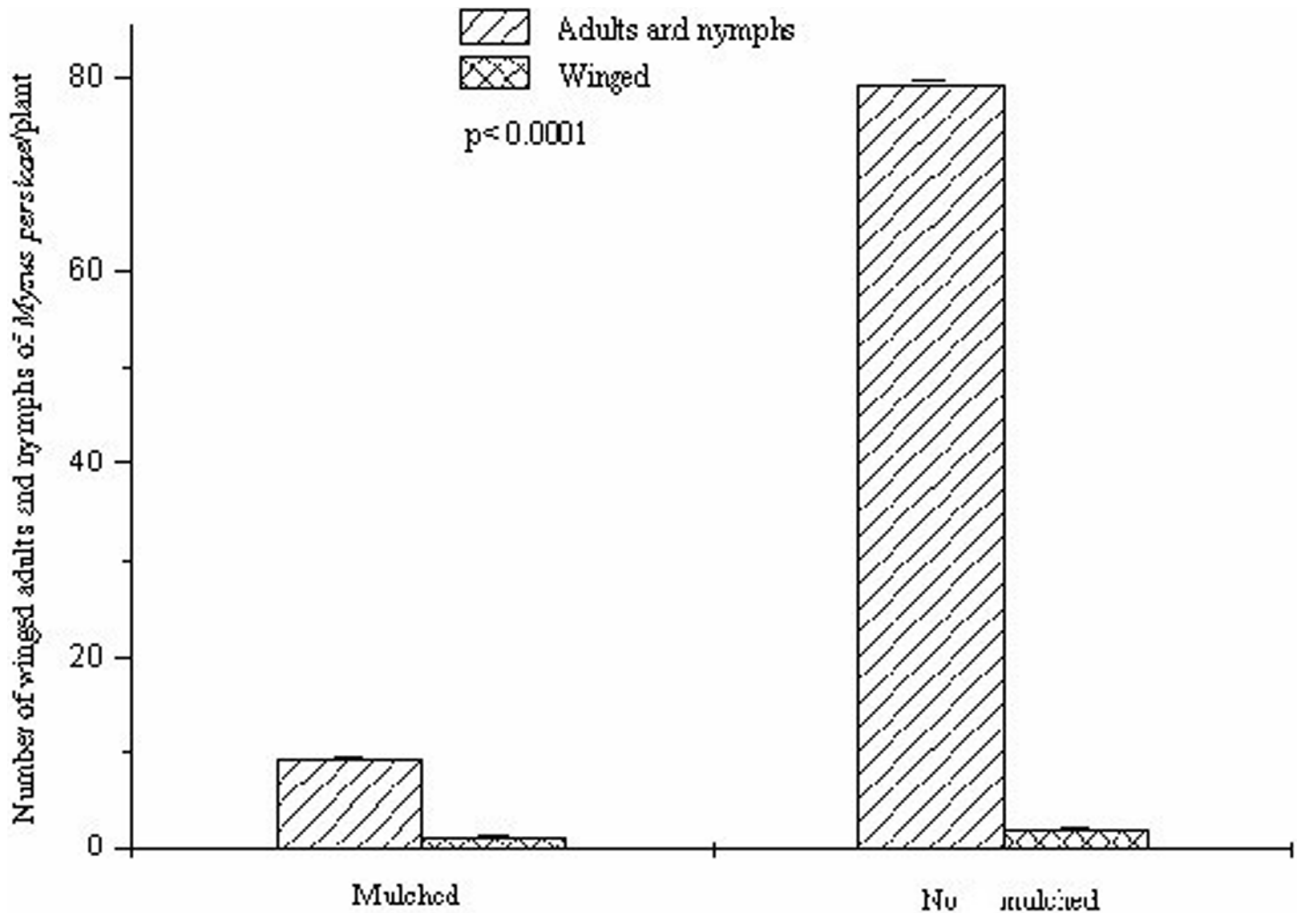
Number of winged adults+nymphs of green peach aphid, *Myzus persicae* (Hemiptera: Aphididae) on kale, *Brassica oleracea* var. *acephala* (Brassicaceae) leaves on mulch and no mulched plots after 15 days of infestation. Poisson error and χ2 of Pearson to correct over dispersion followed by an F test at 5% probability.

The mulch reduced the population growth of *M. persicae* with a lesser number of nymphs, adults (F = 35.62; d.f. = 1.8; p≤0.0001) and winged individuals (F = 8.12; d.f = 1.8; p≤0.03) in the mulched plots 15 days after the inoculation with the winged insect (Fig. 4). In all, 31 adults and nymphs and 1 winged individual were found per colony of *M. persicae* 15 days after the inoculation, with a temperature of 33.8°C in the mulched plots. This value was 318 adults and nymphs and 3 winged adults per colony over the same 15 days in the no mulched plots which had a temperature of 28.9°C.

## Discussion

The temperature increasing in the plots by the mulch may explain the reduced number of arriving winged *M. persicae* on the kale plants as reported for the lower populations of *Aphis* sp. (Hemiptera: Aphididae), *Myzus* sp. and silverleaf whitefly, *Bemisia argentifolii* Bellows & Perring, 1994 (Hemiptera: Aleyrodidae) on watermelon, *Citrullus lanatus* (Thunb.) Matsum. & Nakai (Cucurbitaceae) and corn, *Zea mays* L. (Poaceae) with the polyethylene mulch which was attributed to raising the soil temperature and plant growth besides light reflectance [32]–[34]. This confirms that the mulch can decrease the aphid immigration due to the temperature increase with a negative impact on the aphid populations by slowing down the development and reducing the fecundity [19], [33]. Mulch obtained from a crop of sunn hemp, *Crotalaria juncea* L. (Fabaceae) or weeds (original weed cover) acts as physical barrier to lesser cornstalk borer, *Elasmopalpus lignosellus* Zeller, 1848 (Lepidoptera: Pyralidae) around host plants of bush bean, *Phaseolus vulgaris* L. (Fabaceae) in Florida, USA [35]. High temperatures can be harmful to the developing embryos and thus, reduce the population growth in subsequent generations [36], [37].

The indirect effect of the mulch on the *M. persicae* immigration to the kale plants may have induced this aphid to move to the no mulched plots. This result suggests that the differences in the plant physiology in each environment were maintained for at least seven days. On the other hand, the effectiveness of these characteristics (higher plants and better leaf development) to repel the winged aphids is lower than the mulch effect. Mulching can improve the physical and chemical properties of the soil and favors plant growth [38], [39]. The maintenance of adequate soil conditions is important to assure satisfactory crop growth and high yields [8], [40] with a good impact on the insect pests [41], [42]. Plants cultivated on the mulched plots were more vigorous and with higher biomass which could allow more effective defense mechanisms. This probably includes the production of semiochemicals which repel the aphids, thus resulting in lower *M. persicae* populations [4], [43], [44]. This is due to the humidity maintenance, higher soil temperature, better weed control and mineral nutrient increase in the plants in the mulched plots [45], [46]. The use of cover crops and mulches are convenient non-chemical methods for managing some insect pests and weeds. The numbers of Cicadellidae (Hemiptera), Formicidae (Hymenoptera), Orthoptera, and small-bodied plant-feeders (aphids, Aphidoidea; thrips, Thysanoptera, and whiteflies, Aleyrodidae; Hemiptera) were higher in control (no mulched) or cowpea, *Vigna unguiculata* (L.) Walp. (Fabaceae) plots than several of the other mulch treatment plots (sunn hemp mulch; sorghum-sudangrass mulch, *Sorghum* × *drummondii* (Nees ex. Steud.) Millsp. & Chase (Poaceae), and pine bark nuggets, *Pinus* sp., Pinaceae) in Florida, USA, possibly because weed levels were higher in control and cowpea plots [47]. Fire ants, *Solenopsis* spp. (Hymenoptera: Formicidae) were more abundant where there was mulched cover and are important predators of weed seed and pest insects in killed cover crop plots and that cover crop mulches in summer pepper (*Capsicum annuum* L. cv. ‘Camelot’, Solanaceae) and fall collard (*Brassica oleracea* L. cv. ‘Champion’, Brassicaceae) production are potentially viable alternatives to black plastic mulch and soil fumigation in South Carolina, USA [48].

The indirect effect of the mulch on *M. persicae* immigration was not related to the macronutrient content of the kale leaves and no negative relationship between the aphid landing and expected K content of the plant was found. Nutrients make these plant tissues more resistant and with thicker cellular walls which could affect the feeding preference and increase the resistance to the insects [49], [50]. The UV radiation could increase the tannin accumulation in the lumen and the phenols in the epidermal cells. These factors make the epidermal cells thicker, although with a smaller mesophyll area, suggesting that more carbon is allocated as a protective mechanism with the lower photosynthetic rate [51]. This indicates that the occurrence of these factors and the reduced number of winged aphids on the plants grown or maintained in the mulched plots may be caused by an indirect effect of the UV light reflectance [4], [52]. The inorganic mulch reflects the short-wave UV light [53], which confuses and repel the incoming winged aphids, thus reducing their incidence on the plants [11]. Although the impact of the short-wave light to repel the whiteflies was not conclusive, these insects may respond similarly to those of the winged *M. persicae* approaching the reflective (Al) surfaces [54]. The mulch can influence the concentrations of the carbohydrates and soluble sugars in the kale plants [55], which may have reduced the number of aphids probably due the production of semiochemicals.

The reduction in the *M. persicae* population by the mulch helps to explain the effect of light reflectance. This parameter was not evaluated. However, the UV was responsible as seen by the lesser number of aphids landing on the kale plants [56] and the temperature increased in the cantaloupe, *Cucumis melo* var. *cantalupensis* Naudin (Cucurbitaceae) and tulip, *Tulipa* sp. (Liliaceae) plants on the mulched soil [54]. Temperatures between 25–30°C increased the population growth of the cotton aphid, *Aphis gossypii* Glover, 1887; the turnip aphid, *Lipaphis erysimi* Kaltenbach, 1843 (Hemiptera: Aphididae) and the *M. persicae* on the cotton, *Gossypium hirsutum* L. (Malvaceae) and citrus, *Citrus* sp. (Rutaceae) [57], [58], but temperatures above 30°C reduced their populations [59]. This shows that the higher temperatures in the mulched plots, probably, may have reduced the population growth of *M. persicae* during the 15-day period. Besides, the temperature influences the synthesis of the acyclic polyol (mannitol), common in most animals, but registered only for a few insects [60]. The healthy polyols also protect the proteins against denaturation at high temperatures [61]. The aphids may adapt to the high temperatures by accumulating the polyhydric alcohols in their hemolymph, as observed for mannitol in the greenflies (Hemiptera: Aphidoidea) [62]. The synthesis of these compounds is stimulated by high temperatures. They are not excreted by the aphids; they accumulate in the hemolymph and thus act as a thermo protective mechanism [62]. However, the aphids may not synthesize the mannitol due to the high energy expense and for this reason show a preference for plants with lower temperatures.

The rice-straw mulch decreased the number of winged aphids landing on the kale plants and the growth of this insect colony, which concurs with the lower aphid populations and the reduced incidence of the aphid-borne viruses on the wheat, *Triticum aestivum* L. (Poaceae) and rice plants on mulched soil [4], [63], [64]. The incidence of the *Cucumber mosaic virus* (CMV) *Cucumovirus* (Bromoviridae) on the narrow-leafed lupins, *Lupinus angustifolius* L. (Fabaceae) and the *Bean yellow mosaic virus* (BYMV) *Potyvirus* (Potyviridae) on cereal straw was lower than in the bare soil in Australia. This was explained by the lower occurrence of the virus with a decreasing incidence of the winged aphids landing [65]. The increasing UV reflectance of the backgrounds decreased *Aphis* spp. and *M. persicae* landing on the *B. brassica* plants with a negative correlation of this parameter with the aphid numbers [11]. Straw mulch, therefore, reduced the populations and the numbers of this aphid on the green traps want confirms results obtained in potato [66] and faba beans, *Vicia faba* L. (Fabaceae) [67].

## Conclusions

The lower number of winged *M. persicae* landing and the better vegetative development of the kale plants may be explained by the higher temperature in the mulched plots, as well as to the physiological changes of the kale plants. Besides, the soil covering may reduce the winged *M. persicae* to find the kale plants. The strategy using rice-straw mulch presents a potential to be used in integrated aphid management in the kale plantations.
